# Bike helmets prevent pediatric head injury in serious bicycle crashes with motor vehicles

**DOI:** 10.1186/s40621-020-00249-y

**Published:** 2020-06-12

**Authors:** Stephen J. Strotmeyer, Christopher Behr, Anthony Fabio, Barbara A. Gaines

**Affiliations:** 1 Department of Pediatric General and Thoracic Surgery, UPMC Children’s Hospital of Pittsburgh, 4401 Penn Avenue, Pittsburgh, PA 15224 USA; 2 University of Pittsburgh School of Public Health, 4420 Bayard Street, Suite 600, Pittsburgh, PA 15213 USA

**Keywords:** Bicycle injuries, Bicycle helmets, Head injury, Injury severity, Injury prevention

## Abstract

**Background:**

Approximately 75% of all bicycle-related mortality is secondary to head injuries, 85% of which could have been prevented by wearing a bicycle helmet. Younger children appear to be at greater risk than adults, yet helmet use is low despite this risk and legislation and ordinances requiring helmet use among younger riders. We sought to determine whether bicycle helmets are associated with the incidence and severity of head injury among pediatric bicyclists involved in a bicycle crash involving a motor vehicle.

**Methods:**

We performed a retrospective review of patients age ≤ 18 years hospitalized at a level I pediatric trauma center between January 1, 2008, and December 31, 2018. Data were abstracted from the institutional trauma registry and electronic medical record. International Classification of Diseases 9th and 10th editions and external causes of injury codes were used to identify MV related bicycle crashes and determine the abbreviated injury severity (AIS) for head injury severity. Injury narratives were reviewed to determine helmet use. We calculated the incidence of head injury from bicycle vs. MV crashes utilizing descriptive statistics. We analyzed the risk and severity of injury utilizing univariate and multivariate logistic regression.

**Results:**

Overall, 226 bicyclists were treated for injuries from being struck by a MV. The median age was 11 (interquartile range (IQR): 8 to 13) years. Helmeted bicyclists (*n* = 26, 27%) were younger (9.4 years versus 10.8 years, *p* = 0.04), and were less likely (OR 0.21, 95% CI 0.09 to 0.49) to be diagnosed with a head injury compared to unhelmeted bicyclists (*n* = 199). Of those with a head injury, helmeted bicyclists were less likely (OR 0.57, 95% CI 0.11–2.82) to sustain severe or higher injury using AIS. When adjusting for demographics (age, sex, race) and injury severity, helmet use predicted a reduction in head injury (OR 6.02, 95% CI 2.4–15.2).

**Conclusions:**

Bicycle helmet use was associated with reduced odds of head injury and severity of injury.. These results support the use of strategies to increase the uptake of bicycle helmets wearing as part of a comprehensive youth bicycling injury prevention program.

## Background

Childhood injuries continue to be a major United States (U.S.) public health burden, both economically and as a source of morbidity and mortality (Butchart et al. 2008). Of all childhood injuries, unintentional injuries have been reported as the leading cause of premature mortality (Rezendes 2006). When looking at injuries associated with consumer products, bicycle-related injuries rank second as a cause of injury, behind only motor vehicles (MV) (United States Consumer Product Safety Commission n.d.). Bicycling continues to be a popular recreational activity and a mode of transportation for many children, which can carry considerable risk for injury based on those exposures. Riding in environments deemed unsafe has previously been shown to increase the risk of serious injury, particularly in motor vehicle collisions (MVC) (Rivara et al. 1997). Further research claimed roughly 75% of all bicycle-related mortality is secondary to head injuries, and that 85% of those injuries could have been prevented by simply by wearing a bicycle helmet (Brainline: All about brain injury and PTSD n.d.). Younger children appear to be at greater risk of head injuries than adults, yet despite legislation and ordinances requiring helmet use among younger riders, helmet use has reportedly remained low (Schroeder and Wilbur 2013). In theory, helmets would be inherently protective against head injury, and establishing the real-world effectiveness of helmets involved in MVC resulting in pediatric traumatic injuries is important. Previous reports found bicycle helmets more effective in single bicycle crashes than in collisions with motor vehicles (Høye 2018) A Cochrane review of controlled studies evaluating the effect of bicycle helmets on injuries found helmets provide up to a 63–88% reduction in the risk of head, brain, and severe brain injury for cyclists of all ages (Thompson et al. 1999).

As those studies looked at all bicycle-related crashes, often involving a single bicyclist or a fall from a bicycle, as well as adult riders, it is difficult to determine how effective bicycle helmets are at reducing head injuries among children when involved in an MVC. The objective of this study was to determine whether bicycle helmets are associated with decreased incidence and severity of head injury among pediatric bicyclists involved in a bicycle crash with an MV.

## Methods

We performed a retrospective medical record review of patients aged ≤18 years old hospitalized for bicycle vs. MV collision injuries at a level I pediatric trauma center between January 1, 2008, and December 31, 2018. Patients from the trauma registry were identified using International Classification of Diseases, Ninth & Tenth Revisions, Clinical Modification (ICD-9-CM & ICD-10-CM) external-cause-of-injury codes (E-codes), depending on data year, since ICD-10-CM was the replacement for ICD-9-CM, effective October 1, 2015 (International Classification of Diseases, Tenth Revision, Clinical Modification (ICD-10-CM) n.d.). The ICD-9-CM E-code E813.6 (MVA Traffic, Collision w/ Other Vehicle - Pedal Cyclist), the ICD-10-CM codes V13.4-.9XXA (Pedal cycle driver injured in collision with a car, pick-up truck or van in a traffic accident, initial encounter) and V14.4-.9XXA (Pedal cycle driver injured in collision with heavy transport vehicle or bus in a traffic accident, initial encounter), and medical record injury narratives were used to identify bicycle crashes in traffic with an MV, as well as documented helmet use. Further confirmation to identify bicycle crashes in traffic with a motor vehicle was performed by scanning the mechanism of injury narratives. Helmet use was documented by trauma registrars who compiled data from multiple sources of the medical record, including Emergency Medical Service (EMS) trip sheets, Emergency Department (ED) Notes, trauma notes, and case management notes.

The institutional trauma registrars were trained to perform a daily census of all trauma credentialed floors and actively search for patients with ICD-9/10-CM traumatic injury codes to capture all admitted trauma patients. Medical record data were abstracted and uniformly entered electronically in Digital Innovations Collector/CV-4, a software template that includes an ICD-9/10 Tri-coder. Inter-rater reliability checks were run monthly for many of the elements collected within the database.

Data abstracted from the institutional trauma registry and electronic medical record included demographics, clinical information, anatomic diagnoses, outcomes, and injury severity. Head injury severity was determined using the Abbreviated Injury Scale (AIS©), an anatomically based, consensus derived, global severity scoring system that classifies an individual injury by body region according to its relative severity on a 6-point scale (1 = minor and 6 = maximal). AIS is the basis for the Injury Severity Score (ISS) calculation of the multiply injured patient (Abbreviated Injury Scale (AIS) - Overview 2019). These scores provided standardized injury descriptor capabilities and injury severity assessment, important in clinical trauma management and epidemiological study.

These data were analyzed utilizing descriptive statistics and univariate/multivariate logistic regression. We examined demographics using mean, median, and standard deviation and used binary logistic regression to determine if outcome was impacted by demographics. The data were analyzed using IBM SPSS Statistics v25 (Armonk, NY) (IBM Corp n.d.). Our study was approved by the University of Pittsburgh Medical Center Institutional Review Board (PRO18100096).

Multivariate analysis was done using multiple logistic regression to assess the relationship between helmet status and head injury and the level of head injury severity, as measured by the AIS. The AIS outcomes was dichotomized as severe (4), critical (5) or maximal (6) vs AIS < 4 (Abbreviated Injury Scale (AIS) - Overview 2019). Backward stepwise multivariable logistic regression with a *P*-value < 0.05 was used to identify clinical factors independently associated with the risk of head injury status or head injury severity following an MVC. Variables entered in the first step of this analysis were all those that showed a univariable association with the risk of head injury status or head injury severity, with a *P*-value < 0.10, as well as the age (continuous variable), sex, and race of the patient. Helmet status (*p* < 0.01) retained a statistically significant, independent association with the risk of head injury status or head injury severity at the last step of the backward stepwise process. These variables formed the final multivariable model.

## Results

Looking at the 11 years of trauma registry data, we identified 226 children who were treated for injuries sustained from being struck by a motor vehicle in traffic while riding a bike. The median age of the children treated was 11 years (interquartile range (IQR): 8 to 13). Males made up 83% (187/226) of the patients seen. The patient’s race was recorded as 71% white, 25% black and 4% other or unknown, which was reflective of the catchment area. The median overall injury severity score (ISS) was moderate at 9 (IQR 5–12). Hospital length of stay was a median of 2 days (IQR 1–3), and no appreciable time was spent in the Intensive Care Unit (ICU) or on a ventilator.

Helmets were reportedly worn by 27 (12%) of the patients. The only significant difference observed in demographics when comparing helmeted bicyclists (*n* = 27) to unhelmeted bicyclists (*n* = 199) was age. Helmeted bicyclists were slightly younger (9.4 years versus 10.8 years, *p* = .036) (Table 1).

**Table 1:** Patient demographics by helmet wearing status among 226 bicyclists treated for traumatic injury, 2008–2018

Variable	Helmeted (*n* = 27)	Not helmeted (*n* = 199)
Age*	9 years (Median; IQR 7–13)	11 years (Median; IQR 9–13)
Sex		
Male	23 (12.3%)	164 (87.7%)
Female	4 (10.2%)	35 (89.8%)
Race		
White	23 (14.4%)	137 (85.6%)
Black	4 (7.1%)	52 (92.9%)
Other/Unknown	1 (11.1%)	8 (88.9%)

*Significant at *p* < 0.05

Unadjusted logistic regression analysis showed helmeted bicyclists were 78.6% less likely (OR 0.21, 95% confidence interval (CI) 0.09 to 0.49) to be diagnosed with a head injury compared to unhelmeted bicyclists (Table 2). Further, of those patients sustaining a head injury (*n* = 169, 74.8%), helmeted bicyclists were 43% less likely (OR 0.57, 95% CI 0.11–2.82) to sustain an injury classified as severe (4), critical (5) or maximal (6) under the Abbreviated Injury Scale (AIS) classification (IBM Corp n.d.).

**Table 2:** Head injury status by helmet wearing among 226 bicyclists treated for traumatic injury, 2008–2018

	No head Injury 57 (25.2%)	Head injury 169 (74.8%)	Total 226 (100%)
No Helmet	42 (21.1%)	157 (78.9%)	199 (88.1%)
Helmet	15 (55.5%)	12 (44.5%)	27 (11.9%)

(OR 0.21, 95% CI 0.09 to 0.49)

Multivariate logistic regression examined the relationship between helmet status and head injury and the level of overall injury severity. When adjusting for age (OR 0.99, 95% CI 0.90–1.08) and injury severity (OR 0.91, 95% CI 0.86–0.96), helmet use predicted an 83.4% reduction (OR 6.02, 95% CI 2.4–15.2) in head injury (Table 3).

**Table 3:** Logistic regression analysis of factors used to differentiate between 226 helmeted and unhelmeted bicyclists treated for traumatic injury, 2008–2018

Predictor	Odds ratio	95% Confidence interval	***p***-value
**Age in years**	**0.99**	**0.90–1.08**	**0.77**
**Helmet Status (Yes vs. No)**	**6.02**	**2.41–15.15**	**< 0.000**
**Severity (Severe vs. Not Severe)**	**0.91**	**0.86–0.96**	**0.002**

## Discussion

In support of existing literature, we found helmeted bicyclists involved in MVCs were significantly less likely (78.6%) to be diagnosed with a head injury compared to unhelmeted bicyclists. When a head injury was diagnosed, helmeted bicyclists were significantly less likely (44%) to sustain a severe head injury. Since age was lower among the helmeted compared to unhelmeted bicyclists (9.4 years vs. 10.8 years; *p* < .05) we adjusted for that demographic covariate, helmet status, and injury severity. Our multivariate regression model found helmet use predicted a statistically significant reduction (83.4%) in head injury.

Reducing bicycle-related head injuries and fatalities remains an area for continued prevention efforts to translate research that demonstrated protective effects of bicycle helmets into increased usage. Bicycle helmet safety legislation is associated with decreased fatalities among children riding a bike (Meehan III et al. 2013). Wearing a helmet when riding a bike has been reported as the single most important safety device for decreasing a lower likelihood of head injury (Mehan et al. 2009). Despite helmet laws in 22 states and over 200 additional local laws, many of these injuries still occur while riding a bicycle but have the potential to be preventable through appropriate helmet use (Bicycle Helmet Safety Institute n.d.).

Earlier evaluations summarized the protective effects of bicycle helmets on preventing injuries (providing up to a 63 to 88% reduction in the risk of head, brain, and severe brain injury), for bicyclists of all ages and all types of crashes (Thompson et al. 1999). Our study sought to specifically select pediatric bicycle-related crashes with motor vehicles, theoretically representing the most energy transfer, or high impact collisions resulting in traumatic injury. We found similar benefits of helmet use when looking specifically at children involved in collision with motor vehicles. The results of our study support the use of strategies to increase the uptake of bicycle helmets as part of a comprehensive youth bicycling injury prevention program. As part of those programs, stressing the statistically significant protective effects of wearing a helmet when involved in MVC (arguably the worst situational exposure), in addition to the single-bike crashes or falls, may be a way to communicate real-world effectiveness.

In another population-based study of pediatric bicycle-related head injuries, a small subgroup analysis noted those with severe outcomes, such as intensive care unit admission or death, were patients whose primary injury mechanism was being struck by a motor vehicle. Further, of the youth bicyclists presenting with a head injury, that group was less likely to have worn a helmet, and more likely to undergo CT scans and X-rays when compared to helmeted riders (Kaushik et al. 2015). Motor vehicle involvement increased the risk of serious injury requiring hospitalizations among children treated in emergency departments (McAdams et al. 2018).

There were some limitations in our study. Obvious imperfections of chart review as a data collection method include completeness and accuracy. Despite best efforts by the trauma registrars, the potential for incomplete documentation and the inability to elicit specific information, notably the identification of helmet use, exist. While exhaustively reviewing the medical records (including notes fields from multiple sources), it is possible the team was unable to identify all instances of helmet wearing, particularly if providers failed to report that status in the medical record. While we intentionally sought to identify patients in the trauma registry, which represents the most severe cases of bicycle-related injury resulting from MVC, we would miss those treated and discharged from the Emergency Department (ED). Additionally, we would not capture fatalities of bicyclists who were not transported to our hospital. Our data did not include information on the crash specifics, including speed or vehicle type involved with the collision with the patients. Since we relied on retrospective data with ICD-9/10-CM coding, patient misclassification may have occurred, leading to either over- or under-reporting.

## Conclusion

Children who wore a bicycle helmet use had reduced odds of head injury in our population of children hospitalized after a bicycle vs MV collision. Head injuries were reported in 75% of the patients, yet the severity of the head injury was reduced among the helmeted children when compared to the unhelmeted group. The reduced risk of serious head injury should help drive educational and psychosocial-based interventions to promote increased rates of helmet wearing among youth bicyclists. There is good evidence that helmet legislation is effective at the population level, by increasing use and decreasing head injury once implemented (Huybers et al. 2017; Macpherson and Spinks 2007). A comprehensive approach combining education, awareness and enforcement of helmet use should not only increase helmet use but should additionally address the behaviors of both riders and drivers especially within traffic.

## Data Availability

Study data for the research resides at the Benedum Pediatric Trauma Program in the Division of Pediatric General and Thoracic Surgery at UPMC Children’s Hospital of Pittsburgh, University of Pittsburgh.

## Abbreviations

AIS: Abbreviated Injury Scale
CDC: The Centers for Disease Control and Prevention
CI: Confidence interval
ED: Emergency Department
EMS: Emergency Medical Services
ICD: International Classification of Diseases
ICU: Intensive Care Unit
IQR: Interquartile range
ISS: Injury Severity Score
MV: Motor vehicle
MVC: Motor vehicle collision
OR: Odd ratio
